# Multi-center investigation of cardiac diffusion tensor imaging in healthy volunteers by the Society of Cardiovascular Magnetic Resonance Cardiac Diffusion Special Interest Group NETwork (SIGNET)

**DOI:** 10.1016/j.jocmr.2025.101948

**Published:** 2025-08-25

**Authors:** Irvin Teh, Kévin Moulin, Pedro F. Ferreira, Julie Absil, Maryam Afzali, Peter Agger, Behnaz Akbari, Anthony H. Aletras, Satoru Aono, Charles Benton, Suryava Bhattacharya, Pierre Croisille, Yves De Bruecker, Erica Dall’Armellina, Daniel B. Ennis, Carl Glessgen, Anna Glinska, Sandra Haltmeier, Ariel Hannum, Erik Hedström, Tawfik Hussein, Sarah Jones, George Joy, Karen Kettless, Won Yong Kim, Sebastian Kozerke, Julie Magat, Raja Muthupillai, Reza Nezafat, Sonia Nielles-Vallespin, John Oshinski, Valéry Ozenne, Dudley J. Pennell, Roderick Pettigrew, Iain Pierce, Betty Raman, Agnieszka Sabisz, Jürgen E. Schneider, Janet H. Sherman, Abhishek Shetye, Rolf Symons, Philippe Thoma, Thomas Treibel, Satonori Tsuneta, Jean-Paul Vallee, Niels Vejlstrup, Magalie Viallon, Christopher Nguyen, Andrew D. Scott, Christian T. Stoeck

**Affiliations:** a Leeds Institute of Cardiovascular and Metabolic Medicine (LICAMM), University of Leeds, Leeds, UK; b Department of Cardiology, Boston Children's Hospital, Harvard Medical School, Boston, Massachusetts, USA; c Royal Brompton Hospital, Guy’s and St Thomas’ NHS Foundation Trust and National Heart and Lung Institute, Imperial College London, London, UK; d Radiologie, Imagerie par Résonance Magnétique, Hôpital Erasme, Brussels, Belgium; e Cardiff University Brain Research Imaging Centre (CUBRIC), School of Psychology, Cardiff University, Cardiff, UK; f Department of Clinical Medicine, Aarhus University, Aarhus N, Denmark; g Boston University School of Medicine, Boston, Massachusetts, USA; h Clinical Physiology, Department of Clinical Sciences Lund, Lund University, Lund, Sweden; i Department of Diagnostic and Interventional Radiology, Hokkaido University Hospital, Sapporo, Japan; j National Heart, Lung, and Blood Institute, National Institutes of Health, Bethesda, Maryland, USA; k Division of Cardiovascular Medicine, Radcliffe Department of Medicine, Oxford Centre for Clinical Magnetic Resonance Research, University of Oxford, Oxford, UK; l CREATIS Laboratory, Univ. Lyon, UJM-Saint-Etienne, INSA, CNRS UMR 5520, INSERM 1294, Saint-Etienne, France; m Department of Radiology, Imelda Hospital Bonheiden, Belgium; n Department of Radiology, Stanford University, Stanford, California, USA; o Cardiovascular radiology unit, Radiology clinics, Geneva University Hospital and University of Geneva, Geneva, Switzerland; p 2nd Department of Radiology, Medical University of Gdansk, Gdansk, Poland; q Institute for Biomedical Engineering, University and ETH Zurich, Zurich, Switzerland; r Wallace H. Coulter Department of Biomedical Engineering, Georgia Institute of Technology and Emory University School of Medicine, Atlanta, Georgia, USA; s Barts Heart Centre, St Bartholomew’s Hospital, London, UK; t Siemens Healthcare A/S, Ballerup, Denmark; u Department of Cardiology, Aarhus University, Aarhus N, Denmark; v CNRS, CRMSB, UMR 5536, IHU Liryc, University of Bordeaux, Bordeaux, France; w Department of Radiology, University of Houston, Houston, Texas, USA; x Department of Medicine, Beth Israel Deaconess Medical Center and Harvard Medical School, Boston, Massachusetts, USA; y Department of Radiology and Imaging Sciences, Emory University, Atlanta, Georgia, USA; z School of Engineering Medicine/ENMED, Texas A&M University and Houston Methodist Hospital, Houston, Texas, USA; aa Institute of Cardiovascular Science, University College London, London, UK; ab Department of Cardiology, Copenhagen University Hospital Rigshospitalet, Copenhagen, Denmark; ac Cleveland Clinic, Heart Vascular Thoracic Institute, Cardiovascular Innovation Research Center, Cleveland, Ohio, USA; ad Center for Preclinical Development, University of Zurich and University Hospital Zurich, Zurich, Switzerland

**Keywords:** Cardiac DTI, Intra-site variation, Inter-site variation, Pulse sequence development, Heart, Myocardium

## Abstract

**Background:**

Cardiac diffusion tensor imaging (cDTI) is an emerging technique for microstructural characterization of the heart and has shown clinical potential in a range of cardiomyopathies. However, there is substantial variation reported for in vivo cDTI results across the literature, and sensitivity of cDTI to differences in imaging sites, scanners, acquisition protocols, and post-processing methods remains incompletely understood.

**Methods:**

SIGNET is a prospective multi-center, observational study in traveling and non-traveling healthy volunteers. The study was initiated by the executive board of the Society of Cardiovascular Magnetic Resonance (SCMR) Cardiac Diffusion Special Interest Group (SIG) as a follow-up to a previous multi-center study on phantom validation of cardiac DTI and a recently published SCMR consensus statement on cardiac diffusion MRI. The study has been developed by the Project Management Committee in consultation with the SCMR cardiac diffusion SIG, which includes international experts in cardiac diffusion MRI. To date, more than 20 international institutions have engaged with the study, including sites that are new to cardiac DTI, making this the largest collaborative effort in the field.

**Discussion:**

SIGNET will provide important information about the key sources of variation in cardiac DTI. This will help rationalize strategies for addressing and minimizing such variation. Harmonization of protocols in this and future studies will underpin efforts to translate cardiac DTI for clinical application.

## Graphical abstract

**Figure:**
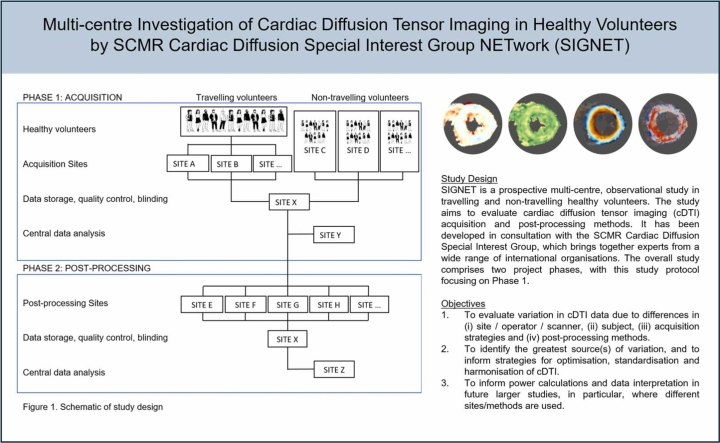

## 1 Background

Cardiac diffusion tensor imaging (cDTI) is an emerging technique for microstructural characterization of the heart. The cardiac function and cardiac cellular micro-organization [1] are highly interconnected. The adult heart comprises more than a billion cardiomyocytes that change orientation in a helicoidal manner from endocardium to epicardium and contract in a highly coordinated manner to generate the cardiac output. Perturbations to the underlying microstructure are an important feature in heart muscle conditions in general. For example, an increase in collagen and scar following remodeling in myocardial infarction, or sheetlet rearrangement in hypertrophic cardiomyopathy and dilated cardiomyopathy.

The microscopic displacement of water molecules due to diffusion [2] is constrained by the presence of cells and by the properties of these cells, such as size, shape, orientation, membrane permeability, and so forth. Thus, by encoding the water diffusion information in the MR image, one can interrogate cardiac microstructure. In cDTI [3], [4], [5], for example, several diffusion encoding directions are sampled and the resulting signal is fitted using a diffusion tensor. From these diffusion tensors [6], several quantitative values can be derived such as the mean diffusivity (MD) that describes the average apparent diffusion, the fractional anisotropy (FA) that describes the eccentricity of the diffusion tensor, the principal directions of diffusivity that represent the voxel-averaged long-axis orientation of the cardiomyocytes (primary eigenvector) and the sheetlet orientation (secondary/tertiary eigenvector). Other metrics can be extracted from the diffusion tensor, such as the helix angle (HA) and sheetlet angle (E2A) that represent the voxel-averaged orientations of cardiomyocytes and sheetlets, respectively, with respect to specific reference planes.

The links between the diffusion tensor and cardiac microstructure have been validated in numerous preclinical studies [7], [8], [9], [10]. Early clinical studies have reported initial diffusion parameters in the pathologic heart. In particular, higher myocardial MD and/or lower FA have been reported in pathologies such as myocardial infarction [11], [12], [13], [14], hypertrophic cardiomyopathy [8], [15], [16], [17], [18], [19], [20], [21], [22], dilated cardiomyopathy [8], [23], aortic stenosis [24], and amyloidosis [25]. Similarly, differences in HA and E2A angles have been reported in these same conditions [8], [13], [23], [24], [25], [26]. This is thought to be related to the underlying changes in the cardiac microstructure.

The field of cDTI remains technically challenging due to factors such as cardiac and respiratory motion, image distortion, and long scan times [27], [28]. Nonetheless, the field has been rapidly expanding over the past years, with publications in cDTI more than doubling over the decade up to 2020 (PubMed search), and whole heart cDTI within clinically feasible scan times looks increasingly likely to be achievable within the foreseeable future. The main challenge thus remains the variability in measurements between vendors, sequences, and post-processing methods.

There exists a wide range of reported cDTI metrics in the literature [29], including reports that are potentially artefactual, which have been previously highlighted [30]. Other potential sources of variation include differences in sites/operators/scanners, acquisition, and post-processing methods. This variation needs to be better understood in terms of precision, accuracy, and intrinsic systematic differences in sensitivity to support clinical validation of the technique and enable clear discrimination of disease-related changes. In a first step, the Society of Cardiovascular Magnetic Resonance (SCMR) Cardiac Diffusion SIG has released a consensus statement [31] to propose a unified approach to acquire, evaluate, and report cDTI findings as basis for further studies.

Earlier work has evaluated reproducibility in a ten-site study in isotropic phantoms [32], and in a two-site study in healthy volunteers [33]. With the present study design proposal, a multi-center collaborative effort is initiated to evaluate cDTI variation due to differences in (i) site/operator/scanner, (ii) subject, (iii) acquisition strategies, and (iv) post-processing methods. The results will guide the interpretation of the cardiac diffusion magnetic resonance imaging (MRI) literature and help to refine strategies for standardization and harmonization of protocols. We propose to study healthy adult volunteers to establish a baseline of variation due to technical (non-pathology-related) factors.

1.1. Objectives

Objectives

1. To evaluate variation in cDTI data due to differences in (i) site/operator/scanner, (ii) subject, (iii) acquisition strategies, and (iv) post-processing methods.
2. To identify the greatest source(s) of variation, and to inform strategies for optimization, standardization, and harmonization of cDTI.
3. To inform power calculations and data interpretation in future larger studies, in particular, where different sites/methods are used.

1.2. Planned outcomes

1. Optimized cDTI acquisition and post-processing methods, refined in collaboration with international expert sites.
2. High-quality cDTI datasets from multiple international sites that will serve as a resource for understanding the variation in cDTI and achieving the scientific aims of the study.
3. Dissemination of cDTI datasets within the study and catalyzation of developments in the field of cDTI.
4. Dissemination of cDTI acquisition and post-processing methods for harmonization of methods to improve prospects of clinical translation.
5. Engagement and community building in the field, and provision of support for sites who are newer to cDTI.
6. Identification of new collaborations amongst and outside of the participating sites.

## 2 Methods

### 2.1 Study design

SIGNET is a prospective multi-center, observational study in traveling and non-traveling healthy volunteers. The study was initiated by the executive board of the SCMR Cardiac Diffusion SIG as a follow-up study to previous work on phantom validation of cardiac DTI [32] and the consensus statement on cardiac diffusion MRI [31]. The study has been developed in consultation with the SCMR Cardiac Diffusion SIG, which brings together experts from a wide range of international organizations.

Sites will participate as either (i) traveling or (ii) non-traveling volunteer sites, not both. cDTI data will be acquired using harmonized MRI sequences and repeated within the same scan session. Data will be anonymized/pseudonymized and sent to the University of Leeds (UoL) for quality control, blinding, and additional anonymisation where necessary. Anonymised data will thereafter be shared by UoL with post-processing sites for central data analysis to ensure consistency in data analysis. Data analysis will include reconstruction of cDTI parameter maps, and assessment of variation in image quality and precision of cDTI parameter maps across sites, repeats, sequences, and subjects. Identical phantoms for each site will be used for data quality checks.

### 2.2 Ethics

The study has been approved by the UoL School of Medicine Research Ethics Committee (MREC 23–027) and the Health Research Authority, UK (IRAS 343837). SIGNET is registered on the 'International Standard Randomised Controlled Trial’ registry: ISRCTN46174869; registration date 12 May 2025; doi:10.1186/ISRCTN46174869. The ethics covers the recruitment and scanning of healthy volunteers by traveling volunteer sites, as well as data sharing and management across traveling and non-traveling volunteer sites. Non-traveling volunteer sites will require their own site-specific ethics that approves recruitment and scanning of healthy volunteers, as well as data sharing for future research. The approved Site Participation Checklist and Declaration has been made available to sites to guide on appropriate wording required for any new ethics or amendments required.

### 2.3 Funding

Participating sites shall be fully self-funded, and cover their own costs associated with the proposed study e.g. scan fees, local data storage, administration, staff costs, phantom. Non-traveling volunteers will be reimbursed by respective recruiting sites for any costs and reimbursement. For traveling volunteers, UoL will be responsible for reimbursement of travel and expenses for traveling volunteers on behalf of traveling volunteer sites. Each traveling volunteer site will reimburse UoL for an equal share of these costs.

### 2.4 Study population

Participants must satisfy the following inclusion criteria:

• Healthy volunteer.
• Sites shall aim to recruit equal numbers of male (N = 6) and female (N = 6) subjects.
• Age 18 to 65 years old.
• BMI 18.5 to 29.9.
• For traveling volunteer study: Ability to travel independently between sites, and to have all scans done within 2 months from the first scan; possession of valid travel documentation.

Participants may not enter the study if any of the following exclusion criteria are known to apply:

• Safety or clinical concerns precluding participation.
• Any history of health conditions that may affect the heart (e.g., hypertension, diabetes, arrhythmias, angina, myocardial, valve and vessel disease, atrial fibrillation, ventricular ectopics).
• Athletes (self-declared).
• Any ongoing cardiovascular medication or other medication that may have secondary effects on the cardiovascular system.
• Pregnancy or breast-feeding, including suspected pregnancy.
• Claustrophobia which limits/prevents participants from remaining in the MRI scanner.
• Inability to lie flat on the scanner table.
• Physical frailty.
• Contraindications to MRI (some pacemakers, intraorbital debris, intraauricular implants, intracranial clips, etc.).
• Those who could be considered to have a particularly dependent relationship with an investigator, e.g. members of staff or students.
• Involvement with the research apart from volunteering.
• Any relevant health conditions precluding safe travel between sites within 2 months of the first scan (for traveling volunteer study).

### 2.5 Sample size

A sample size of N = 12 traveling volunteers enables estimation of limits of agreement in a Bland-Altman plot to within +/−1 standard deviation of the differences between measurements. Based on previous work [33], this would be around +/−10% of the mean for both MD and FA, and applies to the comparison of any two sites, or two scans on the same subject. Inclusion of additional sites and/or subjects would improve precision of the estimate over and above this level, however, the sample size will be limited due to practical reasons. Based on the above, a sample size of N = 12 volunteers was deemed appropriate for both traveling and non-traveling volunteer cohorts, with up to an additional N = 4 as contingency in situations described below.

In exceptional cases, volunteers and/or their data may need to be excluded from the study, and replacement data acquired from other volunteer(s). For consistency, the following describes acceptable reasons for excluding volunteers and/or their data:

• Scan not started or prematurely terminated e.g., due to volunteer wishes, scanner fault, etc.
• Scan protocol not followed e.g., wrong sequence parameters, inclusion/exclusion criteria incorrectly applied.
• Data missing or not acquired.
• Data corrupted or unusable.
• Incidental findings precluding participation.
• Poor electrocardiogram (ECG) signal e.g., evidence of arrhythmia, high prevalence of ectopic beats or missed triggers, etc.
• Poor breath-hold performance where applicable.

In the event of any of the above, sites are advised to try to rectify and repeat the failed acquisitions (within the same scan session) if possible, provided the subject is comfortable to do so. To avoid bias in data, sites are asked not to exclude volunteers or data on the basis of image quality, provided the protocol was followed correctly. Sites are to record and report reasons for exclusion of any volunteers and/or data.

The variation in cardiac DTI due to site/scanner/subject/sequence/post-processing is poorly understood, and therefore, a formal power calculation is not possible. The outputs of this study will inform such power calculations in the future.

### 2.6 Recruitment

Non-traveling healthy volunteers will be identified, approached, and recruited by non-traveling volunteer sites for single MRI scan at one site, in accordance with site-specific ethics.

Traveling volunteers will be recruited and consented for traveling to multiple traveling volunteer sites for single MRI scan at each site. As London is a central location within the participating traveling volunteer sites, it will be most cost-effective for traveling volunteers to be recruited in London. The Royal Brompton Hospital (RBH) has therefore been identified as the central site at which volunteers will be recruited. To this end, healthy volunteers will first be identified via advertisements in newsletters, posters, social media, and/or emails to mailing lists. Potential volunteers who express interest in participating will be sent the enclosed participant information sheet (PIS) and research volunteer checklist.

Prior to consent/scan, volunteers who wish to participate in the study will be asked to complete and return the research volunteer checklist for the study team to assess eligibility. Participants who meet eligibility requirements and are interested in participation, will be invited to provide written consent, which will be received by the study team at RBH. The participant will undergo the MRI scan and the RBH will securely send digital copies of the signed Consent Form and research volunteer checklist to the study team at UoL.

Recruitment will not target potentially vulnerable groups. At the point of first scan, volunteers will be assessed by the respective site for fitness for participation, scanning, and traveling. Any concerns will be discussed with Chief Investigator (UoL) and Site Principal Investigator (RBH), and a decision will be taken to recommend the subject continues/discontinues with the study as would be most appropriate.

For non-traveling volunteer sites, volunteers will be recruited by the individual site, in accordance with the local ethics. Prior to consent/scan, volunteers who wish to participate in the study will be asked to complete and return the research volunteer checklist for the study team to assess eligibility. Participants who meet eligibility requirements and are interested in participation will be invited to provide written informed consent.

### 2.7 Consent

Consent includes:

• single MRI scan at single site (non-traveling volunteer) / multiple sites in the UK and France (traveling volunteers)
• sharing of anonymised/pseudonymised data for future research, as defined by site-specific ethics for non-traveling volunteers or central ethics for traveling volunteers
• traveling between traveling volunteer sites (traveling volunteers only)
• sharing and storing of personal information at the RBH, UoL (Sponsor), and other traveling volunteer sites for study oversight (traveling volunteers only), scan scheduling, safety monitoring, booking travel, and reimbursement
• storing of personal information at the non-traveling volunteer site (non-traveling volunteers),
• permission for reporting of incidental findings to the participants' GP or clinical care team

Where required (e.g. for non-UK sites), traveling volunteers will additionally be consented under respective site-specific ethics outside the UK for scanning healthy volunteers.

Participants will be free to withdraw from the study at any time for any reason. Participants may request to extract, edit, delete, and suspend processing of their personal data. With permission, data collected from volunteers up to the point of withdrawal will be anonymised and used in the research.

Due to the international nature of the traveling volunteer study, traveling volunteers will be expected to have an effective understanding of verbal/written communication in English. For safety reasons (i.e., traveling to multiple sites), subjects with relevant health conditions will not be recruited to the traveling volunteer study.

Any consent taken at sites with non-English documents will be supported by collaborators who can speak English and explain the contents to any volunteers, on the basis of trusted research. Official translation of PIS/consent form will not be mandated.

### 2.8 Data acquisition—in vivo

Any participant undertaking an MRI scan will also complete the standard MRI safety screening on site, before any scan is undertaken.

Prospective sites will be asked to identify 1–2 sequences they wish to use for the study, and to identify which scanners they wish to use, and which software versions apply. Sequence providers, including Cleveland Clinic, CREATIS-Lyon, ETH Zurich, and RBH, will work with sites to disseminate the relevant sequences and provide a standard operating procedure (SOP) for the installation and operation of each sequence. Four sequences will be made available on two vendor platforms as shown in Table 1. Spin echo sequences will use symmetric bipolar diffusion encoding gradients with up to second order motion-compensation [3]. Options for full FOV, reduced FOV, and slice tracking [35] are provided. Stimulated echo acquisition mode (STEAM) sequences will use non-motion-compensated pulsed gradient spin echo (PGSE) monopolar gradients (Stejskal and Tanner, 1965). Sequences have been harmonized to facilitate inter-sequence comparisons. However, some variation exists e.g., use of minimum TE is specified to maximize site-specific gradient performance. A custom diffusion direction scheme is specified comprising three orthogonal directions for the b = 50 s/mm^2^ acquisition and 12 directions [36] distributed over a hemisphere for the b = 400 s/mm^2^ acquisition, with eight repetitions of each direction.

**Table 1:** Harmonized scan parameters for four cDTI sequences on two available vendors: (left to right) Up to M2 motion compensated spin echo (M2SE) with full FOV, M2SE with reduced FOV, M2SE with full FOV and slice tracking, and stimulated echo acquisition mode (STEAM).

			M2SE full FOV		M2SE reduced FOV		M2SE full FOV (Slice Tracking)		STEAM	
			Siemens	Philips	Siemens	Philips	Siemens	Philips	Siemens	Philips
General	Repetition time (TR)	#RR	3	3	3	3	3	3	2	2
	Echo time (TE)	ms	Select minimum. Range: 54 to 107 ms						Range: 22 to 34 ms	
	#satbands		2	2	2	OVS	2	2	0	0
	Fatsat		SPAIR	SPIR	SPAIR	SPIR	SPAIR	SPIR	CHESS	SPIR
	Bandwidth	ms	Select minimum. Range: 1900 to 2500 Hz/px							
	time@60bpm	s	360	360	360	360	360	360	360	240
	Breath-hold		N	N	N	N	N	N	Y	Y
	ECG trigger		Y							
	Trigger time (TT)	ms	= 0.85 * end-systole (max contraction) measured from cine; TT = RR to kzero							
	Software versions available		VE11C/E XA30/31/ 50/51/ 60/61	R5.7.1 / R11.1	VE11C/E XA30/31/ 50/51/ 60/61	R5.7.1 / R11.1	XA20/30/ 31/50/ 51/60/61	R5.7.1 / R11.1	VE11C/E XA50/51/ 60/61	R5.7.1 / R11.1
Geometry	FOV (read)	mm	300	300	300	300	300	300	314	314
	FOV (phase)	mm	300	300	120	120	300	300	118	116
	FOV (phase)	%	100	100	37.5	37.5	100	100	37.5	35.6
	Matrix (read)		128	128	128	128	128	128	112	112
	Matrix (phase)		128	128	48	48	128	128	42	40
	Partial Fourier		6/8	6/8	7/8	7/8	6/8	6/8	None	None
	Reduced FOV		None	None	ZOOM-IT	NCP-Exc	None	None	NCP-Exc	NCP-Exc
	#slices		3	3	3	3	3	3	1	1
	orientation		Mid short-axis slice							
	in-plane resolution	mm	2.3	2.3	2.3	2.3	2.3	2.3	2.8	2.8
	slice thickness	mm	8							
	Slice tracking		N	N	N	N	Y	Y	N	N
	Parallel imaging		GRAPPA	SENSE	None	None	GRAPPA	SENSE	SENSE	SENSE
	Accel factor		2	2	NA	NA	2	2	2	1.8
Diffusion	b_low	s/mm^2^	50							
	b_high	s/mm^2^	400							
	#repetitions		8							
	#dirs (b_zero)		0 (in vivo) / 8 (phantom)							
	#dirs (b_low)		3							
	#dirs (b_high)		12							
	#total_acqs		120 (in vivo) / 128 (phantom)							

*FOV* field of view, *SPAIR* spectral adiabatic inversion recovery, *SPIR* spectral presaturation with inversion recovery, *GRAPPA* generalized autocalibrating partially parallel imaging, *SENSE* sensitivity encoding, *NCP-Exc* non-coplanar excitation [34], *OVS* outer volume suppression [40].

MRI scans will be performed at participating sites using a range of scanners (Philips Healthcare, Best, The Netherlands and Siemens Healthineers, Erlangen, Germany), field strengths (1.5T to 3T), and gradient performance (45 mT/m to 200 mT/m). Appropriate RF coils will be used, typically multi-channel phased array body and spine coils. Acquisitions will be ECG-triggered. Each cDTI scan will take approximately 6 min to acquire based on a heart rate of 60 beats per minute (bpm). Two sequences with single repeats will take about 24 min. The recommended time for the full protocol (below), including scout, cine localizers, and cDTI scans is up to 90 min which should suffice for sites at all levels of experience.

1. Subjects will be first registered on the scanner with a subject ID and dummy date of birth. Real names and dates of birth shall not be used.
2. At the start of the scan, sites shall obtain a multiplane isocentre localizer for slice planning.
3. Two chamber, four chamber, and short-axis views (single-slice only) using breath-hold cine will be acquired as per SCMR-recommended cardiac MRI protocols https://marketing.webassets.siemens-healthineers.com/1800000000013244/bac7ca80ce19/CMR_Users_Guide_B17_1800000000013244.pdf. The timing of end-systole (T_ES_) will be identified as the time from ECG R-wave to maximum contraction of the left ventricle. T_ES_ will be assessed based on the short-axis localizer and four-chamber localizer data and averaged.
4. cDTI data shall be acquired in short-axis view centered between the mitral valve and the apex in the systolic configuration (Fig. 1). For SE sequences, two saturation bands shall be placed around the heart, perpendicular to the short-axis view, covering the majority of subcutaneous chest fat. No saturation bands are applied for the STEAM sequences. The shim volume shall correspond to a tight region around the left ventricle. ECG gating will be employed for cDTI sequences. cDTI scans will be acquired in late systolic phase, by setting the trigger delay such that the trigger time (TT) shall correspond to 85% of T_ES_.

**Fig. 1:**
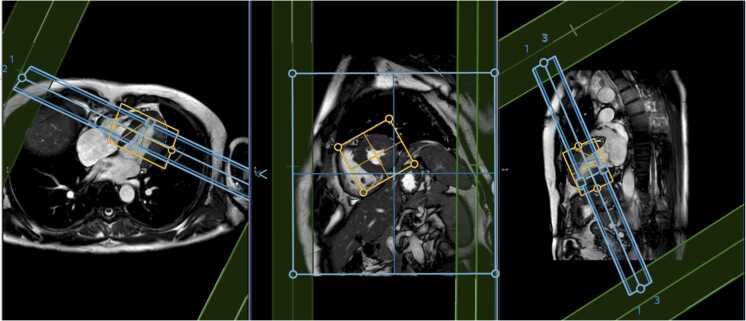
Example planning of full FOV acquisition, showing the slice planning for imaging (cyan), shim (orange), and saturation bands (green). *FOV* field of view
5. Scout cDTI data with minimal numbers of diffusion directions shall be acquired to ascertain image quality. Where distortions are apparent (Fig. 2), the fat shift direction along the phase encoding direction is inverted. Once a suitable FOV has been identified that minimizes susceptibility artifacts, sites will proceed to obtain cDTI data with the sequences specific to each site.

**Fig. 2:**
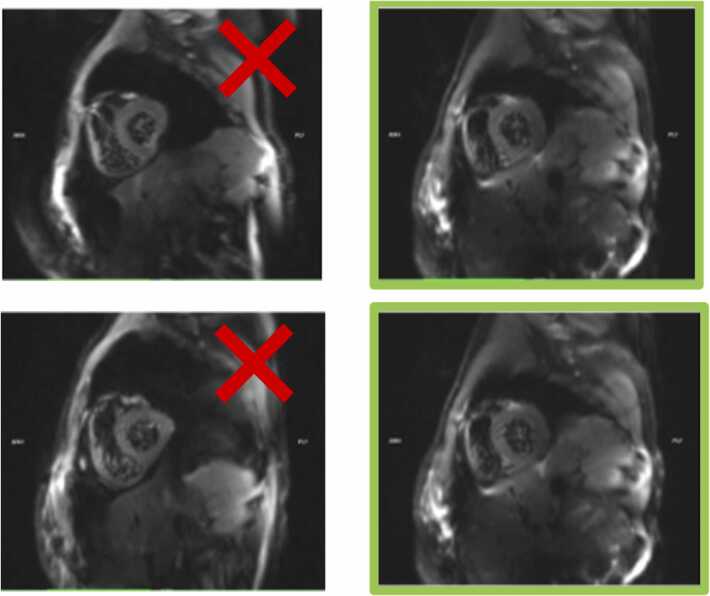
Example of susceptibility artifact (left) near the posterior vein of the left ventricle, modified by adjusting the fat shift direction on Philips scanner
6. Once all scans are completed, subjects shall be removed from the scanner (i.e., step off the scanner bed) and immediately repositioned for a repeat scan. ECG leads should not be removed. Steps 1-5 (including new localizers) to be repeated in order to assess repeatability.
7. DICOM data shall be exported from the scanner in anonymised format as detailed in sequence-specific SOPs. Sites are strongly encouraged to anonymise their data and convert to NIFTI format before sharing with UoL via the Globus platform. Such conversion and anonymisation are mandatory for sites whose site-specific ethics stipulate that only anonymised data may be shared, and optional for sites whose site-specific ethics stipulate that pseudonymised data may be shared. An SOP and a script (https://github.com/ImperialCollegeLondon/cdti_data_export) have been made available to sites for this purpose. Outputs from the script include NIFTI data and associated files, including header (.nii,.json,.bval,.bvec) and helper files including.yaml (and.csv for STEAM).
8. To facilitate central data analysis, data shall be organized and exported in the following folder structure Organisation_Name > Data_type > Sequence_name > Subject_ID > Scan_ID with NIFTI and associated files (or DICOM files) to be stored in the Scan ID folder. Sites shall endeavor to share only NIFTI and associated files, not DICOM data. If conversion to NIFTI is not possible, and sharing of DICOM data is permitted by site-specific ethics, DICOM data may be uploaded. For sites using more than one scanner, this shall treated as a different Organization Name in the directory structure. For Subject IDs, non-traveling volunteer sites to use the naming convention below. Subjects scanned by a given site on multiple scanners shall be given the same subject ID on each scanner. For blinding purposes, traveling volunteer sites will be supplied with site-specific Subject IDs by UoL

**Figure:**
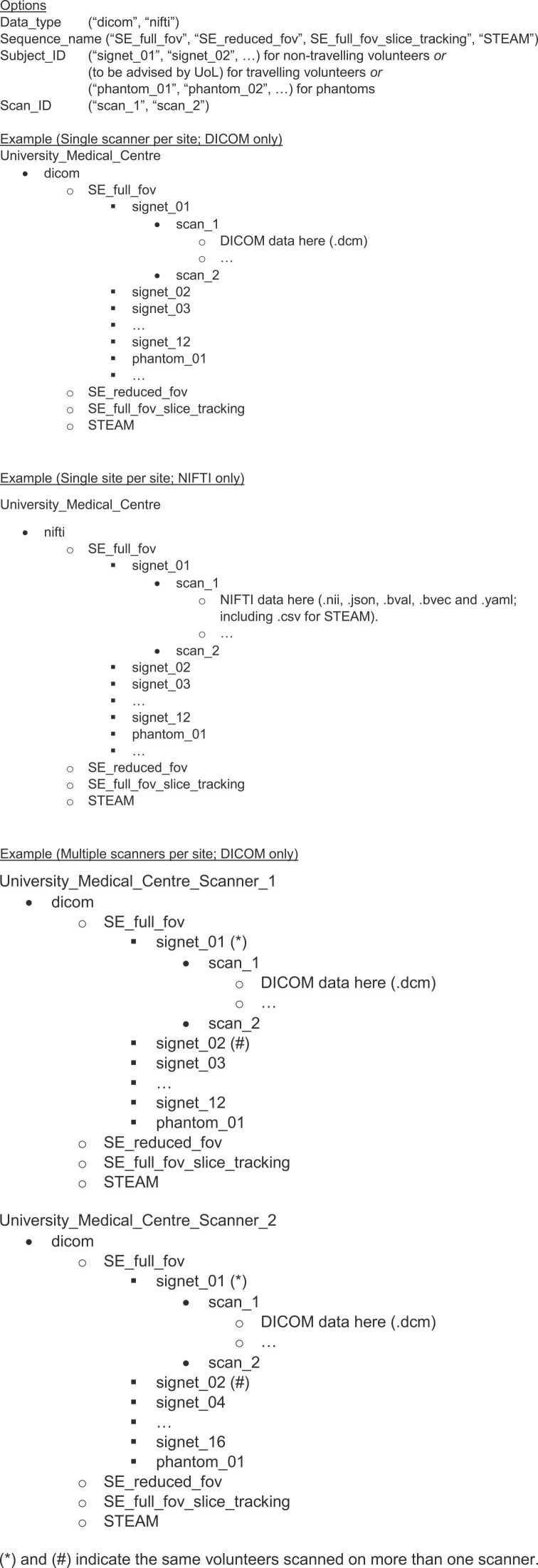
9. Incidental findings will be reviewed and reported at an individual site level, in accordance to local practices.

### 2.9 Data acquisition—phantom validation

**Fig. 3:**
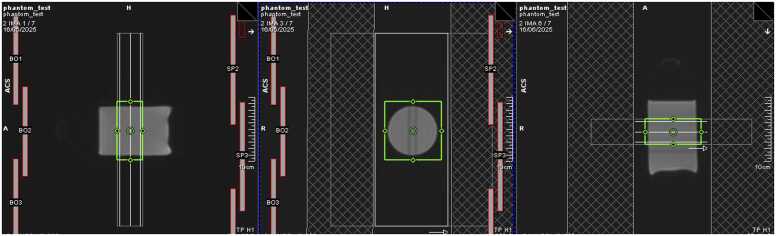
Example scout image of phantom showing the slice planning for imaging (white), shim (green), and saturation bands (hatched)

A sealed bottle of cyclooctane (250 mL or ideally 500 mL depending on availability) shall be used as an isotropic diffusion phantom for validation of MD measured using different sequences. The sequences will be identical to the in vivo acquisition, with the addition of eight non-diffusion-weighted images to facilitate online reconstruction and display of MD maps for user quality checks. Non-diffusion-weighted data shall be removed by the central data processing site in post-processing, such that the in vivo and phantom acquisitions are effectively the same. Additionally, a non-motion-compensated PGSE cDTI dataset will be acquired using vendor product sequences as a reference. The sequence parameters will correspond to the M2SE Full FOV sequence (Table 1), except for the use of non-motion-compensated PGSE diffusion encoding waveforms and a fixed TE of 60 ms across all scanners. A single dataset per sequence will be acquired. The bottle will be oriented vertically in the scanner and scanned in coronal plane to yield a circular cross-section (Fig. 3). ECG simulation of 60 bpm will be activated. Temperature in the scanner bore will be monitored with a lab thermometer. Sites shall verify that MD reconstructed online is within ±10% of expected MD adjusted for temperature [37]. Once satisfied, phantom data and temperature reading will be uploaded to UoL and processed by central data processing sites to (i) validate the process of data export and anonymisation, and (ii) verify that MD values are within ±10% of the temperature-specific MD based on independent reference data [37].

### 2.10 Data management, protection, and confidentiality

Dedicated secure network storage hosted by UoL has been set up, which allows for (i) upload/download of MRI data from participating sites via Globus front-end, (ii) user-defined permissions for creating public/private spaces, and (iii) automatic backup of data. Sharepoint will be used as a means of storage and transferring non-MRI data between sites. Study documents including study protocol, PIS and informed consent form templates, Site Participation Checklist and Declaration, and SOPs for in vivo and phantom scanning will be made available via Sharepoint.

UoL will serve as coordinating center for data management and dissemination. Data received by UoL will undergo checks for anonymisation and undergo further anonymisation as needed, before sharing with central data analysis site(s) who will be blinded to the data.

### 2.11 Data analysis

**Fig. 4:**
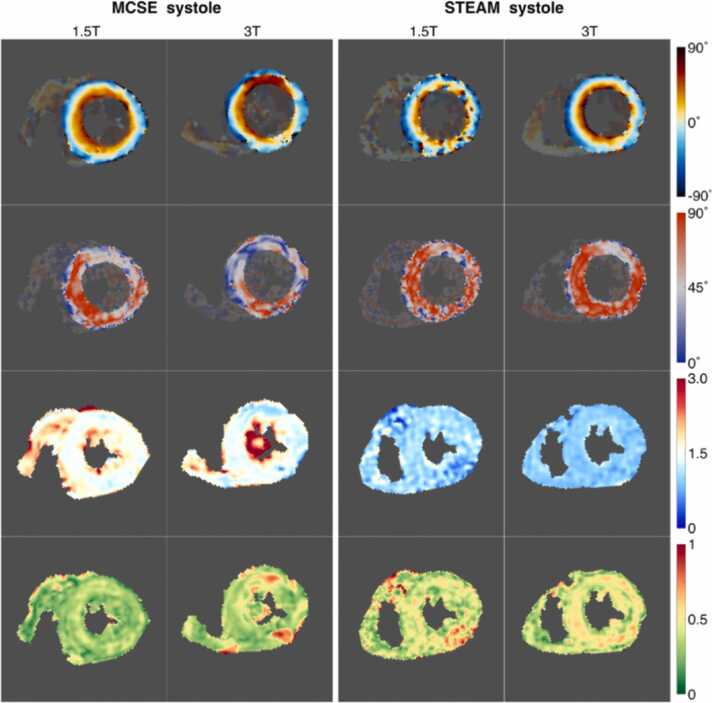
(Top to bottom) Example HA, E2A, MD, and FA maps in a healthy volunteer acquired with M2SE and STEAM at 1.5 and 3T. Adapted from Scott et al. [39]. *HA* helix angle, *E2A* sheetlet angle, *MD* mean diffusivity, *FA* fractional anisotropy, *M2SE* Up to 2nd order motion compensated spin echo, *STEAM* stimulated echo acquisition mode

For consistency in post-processing, cDTI data will be processed and analyzed centrally, with data processing sites blinded to the site and subject information. cDTI data from each scan will undergo the following semi-automated steps: image registration, outlier rejection, weighted or non-linear least squares fitting of diffusion tensor, generation of cDTI maps (MD, FA, HA, E2A; Fig. 4), segmentation into American Heart Association segments, reporting of parameters (e.g., mean, standard deviation, median, interquartile range) at the segment, slice and global levels. For STEAM data, actual b-values will be calculated based on recorded RR-intervals extracted from the image header information and used for fitting diffusion tensors. Signal-to-noise ratio will be assessed based on the voxel-wise standard deviation across repetitions of the same encoding [38]. Repeatability of cDTI results will be assessed by Bland-Altman plots. Data from sites will be grouped according to field strength, pulse sequence, gradient performance, and other relevant parameters, and appropriate statistical techniques will be used to examine group differences.

## 3 Discussion

SIGNET is a prospective multi-center, observational study in traveling and non-traveling healthy volunteers. With more than 20 sites engaged as prospective participants, this is the largest study of its kind. Obtaining baseline measurements in healthy volunteers in such a broad spectrum of scanner and sites is a considerable undertaking that involves creation and dissemination of SOPs and sequences for different vendors, scanners and software versions, as well as ethics and legal agreements to facilitate exchange of IP and data. However, it is expected to provide vital new information on precision and potential bias in cDTI parameters, and to help evaluate the influence of site, scanner, sequence, subject, field strength and other relevant parameters on cDTI. This in turn will help the research community with prioritizing strategies for addressing and minimizing such variation, and support power calculations in future studies. Further, the results from this multi-center study will inform studies in pathologies on the baseline reproducibility achievable by cDTI. Crucially, the understanding gained from the study is expected to support ongoing efforts in harmonization and standardization of cardiac DTI methodologies compatible with a broad range of real-world scenarios, and serve as a milestone and prerequisite to clinical translation of cardiac DTI. The study also provides a large cohort dataset that will be made available for other sites to develop and benchmark their post-processing and analysis frameworks.

## 4 Future work

In a future phase of the study, it is planned to make a subset of anonymised data available to sites as part of a post-processing challenge. Post-processing sites will process the data, reconstruct cDTI parameter maps, and send the reconstructed data to UoL for quality control and blinding. Data will thereafter be shared with a central site for assessment of post-processing performance. This will help to improve understanding of the sensitivity of cardiac DTI parameters to post-processing methods, and help to rationalize and harmonize optimal strategies for post-processing.

## Funding

ADS, PFF, DJP, and SNV are funded by BHF grant RG/F/23/110115; the Engineering and Physical Sciences Research Council grant EP/X014010/1; the Chan Zuckerberg Initiative grant 2024–337787; and Heart Research UK grant NET24–100009. CN acknowledges funding from the National Institutes of Health R01 EB033853; R01 HL151704; R01 HL159010. IT acknowledges funding from the British Heart Foundation, UK PG/19/1/34076. JES acknowledges funding from the Wellcome Trust 219536/Z/19/Z. ST acknowledges funding from JSPS KAKENHI 23K14910 and a Hokkaido Heart Association Grant for Research.

## Author contributions

Study design, planning, sequence optimization, SOP preparation and review of study documentation (A.S., C.N., C.T.S., I.T., K.M., P.F.); pulse sequence development and provision (A.S., C.N., C.T.S., K.M.); setup of data handling pipeline (C.T.S., I.T., P.F.); data storage management, drafting of ethics approval and collaboration agreement (I.T.); initial drafting of manuscript (C.T.S., I.T.); site setup and coordination of local approvals, feedback on study design and SOPs, review and approval of final manuscript (All).

## Declaration of competing interests

The authors declare the following financial interests/personal relationships which may be considered as potential competing interests: Andrew D. Scott, Pedro F. Ferreira, Dudley J. Pennell, Sonia Nielles-Vallespin reports that financial support was provided by British Heart Foundation. Andrew D. Scott, Pedro F. Ferreira, Dudley J. Pennell, Sonia Nielles-Vallespin reports financial support was provided by Engineering and Physical Sciences Research Council. Andrew D. Scott, Pedro F. Ferreira, Dudley J. Pennell, Sonia Nielles-Vallespin reports financial support was provided by The Chan Zuckerberg Initiative. Andrew D. Scott, Pedro F. Ferreira, Dudley J. Pennell, Sonia Nielles-Vallespin reports financial support was provided by Heart Research UK. Christopher Nguyen reports financial support was provided by National Institutes of Health. Irvin Teh reports financial support was provided by British Heart Foundation. Jürgen E. Schneider reports financial support was provided by Wellcome Trust. Satonori Tsuneta reports financial support was provided by JSPS KAKENHI. Satonori Tsuneta reports financial support was provided by Hokkaido Heart Association. Dudley J. Pennell, Andrew D. Scott, Sonia Nielles-Vallespin, Pedro F. Ferreira reports a relationship with Siemens that includes: funding grants. Reza Nezafat reports a relationship with National Institutes of Health that includes: funding grants. Reza Nezafat reports a relationship with Siemens Healthineers that includes: non-financial support. Karen Kettless reports a relationship with Siemens Healthcare that includes: employment. If there are other authors, they declare that they have no known competing financial interests or personal relationships that could have appeared to influence the work reported in this paper.

## Data Availability

To maximize impact and uphold the principles of Open Science, anonymised data acquired by sites will be made available to the research community for future research. Data sharing will be governed by informed consent, end-user licence agreement, collaboration agreement, and site-specific ethics as applicable.
